# Comics as a Medium for Parent Health Education: Improving Understanding of Normal 9-Month-Old Developmental Milestones

**DOI:** 10.3389/fped.2018.00203

**Published:** 2018-07-19

**Authors:** Eduardo D. Rosas-Blum, Hector M. Granados, Brandy W. Mills, Marie Leiner

**Affiliations:** ^1^Department of Pediatrics, Paul L. Foster School of Medicine, Texas Tech University Health Sciences Center, El Paso, TX, United States; ^2^Department of Education, Paul L. Foster School of Medicine, Texas Tech University Health Sciences Center, El Paso, TX, United States

**Keywords:** multimodal literacy, parents, developmental milestones, child development, comics

## Abstract

Multimodal literacy, a product of modern technology, can aid in the recall of simple-to-complex information for both children and adults. Health education information presented using educational comics takes advantage of multimodal formats and designs based on theoretical models of learning. In this study, we utilized a quasi-experimental design with both pre- and post-intervention testing. The parents of every other patient that attended the well-child appointments of their child aged <9 months were invited to participate in this study. Participants were drawn from three pediatric clinics, with a total of 280 parents included in the study. Each parent completed a pre-intervention test consisting of an eight-item questionnaire regarding the developmental milestones of a 9-month-old child. After responding to the questionnaire, the parents received a comic about a 9-month-old child reaching age-appropriate developmental milestones. Four to six weeks after the comics were provided to the parents, they responded to the same questionnaire by phone, which consisted of the same eight questions plus an additional question regarding possible additional uses of the comic. Parents significantly increased their recall of information of developmental milestones when the pre- and post-intervention test results were compared, with a significance of *p* < 0.001 at a 95% confidence level. Additional uses of the comic reported by parents included calling their pediatrician with doubts about their own child's appropriate achievement of milestones, and lending the comics to relatives or friends. The educational comic appeared to assist parents in making meaningful connections between the simplified pictures and the developmental milestones of their child. Comics may provide an alternative for parental education using this multimodal format to explain simple-to-complex issues.

## Introduction

Since the advent of modern technology, the concept of literacy has undergone a process of perpetual transformation (1–3). While the traditional definition of literacy has been confined to the reading and writing of printed text, technology has allowed literacy to become a multimodal entity presented in differing formats, including visual, graphic, and musical communication (4, 5). These complex and varied presentations allow for creative compositions that can effectively communicate information by utilizing meaningful model and symbol systems intended to denote an idea, story, or educational concept.

Undoubtedly, these multimodal presentations can address complex subjects, such as health, disease, and disability to children (6–8). However, adults, particularly those who experience barriers to communication, can also benefit from alternative media formats that embody multimodal characteristics, such as educational comics (9). In a multimodal format, comics depicting a story in a sequence of pictures or cartoons may be helpful to adults with language, education, and/or literacy disparities (10). The pictures and text are not only complementary, but often work in parallel, adding color, space, and words generally written at a fifth- or sixth-grade reading level. The narrative is then simple, brief, and clear, and uses characters to express feelings, thoughts, and actions that enhance the text (9–11).

These specific characteristics are necessary when communicating health information because they provide the setting for viewers to learn simple and complex subjects in a multimodal presentation where modeling can be used to promote behavior modification. Learning and behavioral changes are promoted to serve as a vehicle for personal change using theories of communication applied in the construction of the comic. Under this premises, day-to-day activities may provide a model for observational learning in concordance with Albert Bandura's social learning theory (12). For example, socially desirable and undesirable behaviors along with consequential reinforcement and punishment, respectively, can easily be modeled in the comic, allowing the reader to absorb ideas, values, and experiences from which they can learn (13).

Comics, a unique multimodal educational tool that promotes attention, can be relatable and memorable because of the simplicity of the model presented in the medium. Additionally, comics augment retention and understanding by offering plentiful mental imagery and concise written descriptions. This may be especially helpful in the medical field, where there is often a need to simplify complex information, such as for patients with limited health literacy and/or patients with communication barriers.

While educational comics have been explored by some as a means to educate patients (children or adults) on specific conditions, the role of comics in educating caregivers on normal developmental milestones has not yet, to our knowledge, been analyzed. In this study, we developed an educational comic regarding developmental milestones at 9 months of age and evaluated parental recall of the information as well as additional uses of the information presented in the comic.

## Methods

### Design and setting

This study consisted of a quasi-experimental design with pre- and post-intervention tests. Participants were parents attending their child's 9-month well-baby visit. The study was conducted over a 9-month period at three university-based clinics located in a large metropolitan city. Every other patient was selected and invited to participate in the study (systematic sampling randomization). The population of patients attending these clinics was predominantly Hispanic, a population in which literacy and socioeconomic level are low, according to previous studies (14, 15). All parents/caretakers were asked to complete a pre-intervention eight-question questionnaire. After the pre-intervention questionnaire was completed, parents received a comic including a story about a 9-month-old child reaching the age-appropriate developmental milestones. After 4–6 weeks, participants were contacted by phone to respond to a questionnaire that included the same eight questions as the pre-test as well as an additional question regarding the parents' use of the information presented in the comic.

### Comic

**Figure 1:**
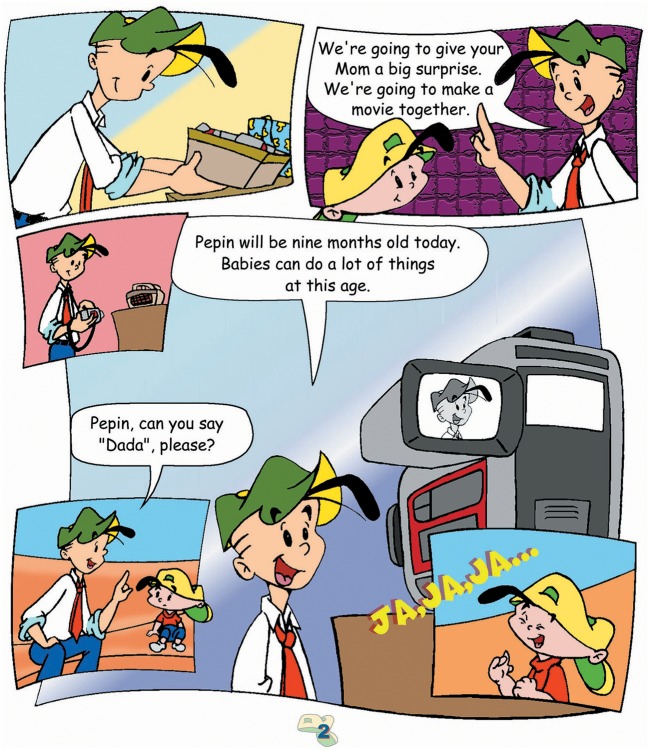
Sample page of nine month old comic used in study.

A multidisciplinary team that included physicians, nurses, a researcher, and graphic designers produced the comic. The comic contained information about developmental milestones appropriate for a 9-month-old child as well as safety guidelines. Developmental milestones were compiled from information available from established medical societies, including the American Academy of Pediatrics, and was provided to all parents and caregivers included in the study free of charge. A story was prepared both in English and Spanish using cartoons portraying a mother and father with a 9-month-old child. In the story, the child and the parents faced day-by-day experiences of child development and life in general. The father is left alone with the child and decides to make a movie with the baby demonstrating the 9-month milestones. The comic used humor to model the responses of the baby that did not necessarily occur when the father was ready to record the movie (Figure 1). At the end of the comic, safety guidelines are mentioned. The story was produced with the intention of conveying the developmental milestones of the child as well as the frustration of the parent who handled the situation with “humor and patience.” The intention was to provide a story where child and parent behaviors are modeled, thereby providing a model for observational learning.

### Survey questionnaires

Two pilot questionnaires were conducted in order to validate the questions and the responses, and the questionnaires were modified according to the information gathered in the pilot studies. Data gathered from the participants in this pilot indicate the questions were clear and comprehensive. The pre-intervention (baseline) included demographic information (age, ethnic origin, gender, and education level) and eight questions related to understanding child developmental milestones at 9 months of age, including gross motor, fine motor, language, intellectual, and social skills. These questions were selected to reflect typical developmental milestones achieved during the 9 month well-child visit. The 4–6-week post-intervention questionnaire included the same eight questions contained in the pre-intervention questionnaire, but were scrambled to reduce the possibility of parents recalling the questions, and possibly biasing the responses.

Sample of questions used:

At 9 months of age, a baby can pick up small items with one hand.
At 9 months of age, a baby can feed himself a cracker or cookie.
At 9 months of age, a baby can walk while holding on to furniture.
Responses included three options: “true,” “false,” and “I do not know.”

Sample size: With an effect size >0.2, a random sample of 90 pairs of measurements will give us an 80% power to state that the mean of the paired differences is significantly different from zero.

### Data and statistical analysis

All analyses were carried out using SPSS for Windows version 19.0, IBM. Descriptive statistics were reported as the mean (SD) for the number of questions answered correctly.

The significance of change in recall of information post-intervention (4–6 weeks after well-baby visit) vs. baseline was examined using a paired *t*-test.

This study was approved by the Institutional Review Board (IRB) of Texas Tech University Health Sciences Center.

## Results

Parents who attended the clinics until we completed 280 participants were invited to participate in this study before their child was 9 months of age. A total of 280 parents completed the pre-intervention questionnaire and were provided with comics. Of 280, 243 (86.8%) were successfully contacted 4–6 weeks after completing the pre-intervention questionnaire. The 37 parents (13.2%) who were not successfully contacted were not included in the study. Descriptive information of the participants is presented in Table 1. Two ethnicity groups were represented, including Hispanic (majority 97%) and white non-Hispanic. Responses in English occurred in almost half of the sample (49.9%). A total of 26.3% of the participants indicated studies above high school.

**Table 1:** Demographic characteristics of sample.

	***N* = 243**
**GENDER**	
Female	226 (93.0)
Male	17 (7.0)
**EDUCATION**	
Elementary	16 (6.2)
Middle	51 (21.0)
High School/GED	97 (39.9)
Technical	16 (6.6)
College	64 (26.3)
**AGE GROUPED**	
18–22	84 (34.6)
23–27	64 (26.3)
28–32	60 (24.7)
33–37	28 (11.5)
>38	7 (2.9)

We observed that the average number of correct responses was smallest at pre-intervention and significantly increased over time. The average (SD) number of correct answers was 2.53 (.91) at pre-intervention and 6.93 (.88) at 4–6 weeks post-intervention (*t* = −73.57, df = 242 *P* < 0.001). Significance between the pre-intervention and post-intervention number of correct responses was found even after stratifying by educational level (Table 2). No significant difference was found between the English and Spanish responses (data not presented).

**Table 2:** Paired samples *t*-test comparing pretest and post-test scores by education level.

	***N* = 243**	**Pre-test mean (SD)**	**Post-test mean (SD)**	***t*-value**	***p*-value**
**EDUCATION**					
Elementary	16 (6.2)	2.13 (0.83)	6.27 (0.89)	−15.01	<0.001
Middle	51 (21.0)	2.31 (1.05)	6.78 (0.86)	−35.38	<0.001
High School/GED	97 (39.9)	2.57 (0.92)	7.03 (0.80)	−43.94	<0.001
Technical	16 (6.6)	2.38 (0.81)	7.06 (1.00)	−31.14	<0.001
College	64 (26.3)	2.78 (0.75)	7.02 (0.93)	−39.02	<0.001

Parents were questioned about their use of the information presented in the comic. All but one parent described at least one use of the information presented, which included: (1) telling a friend about child development at 9 months of age (47%), (2) calling their pediatrician about possible developmental delays of their child at an early age (12%), and (3) sharing the material with relatives, including parents or spouses (56%).

## Discussion

Our study indicates that the majority of parents who participated in the study read the comic, shared it with relatives or friends, and learned from the story, resulting in a significant increase in knowledge compared to the pre-study questionnaire results. Parents/caretakers also used the information provided to discuss and share information with their own primary care physician. These results are worth considering because they demonstrate the possibilities of non-conventional models of communication. Comics may prove to be more useful than their traditional role as inexpensive entertainment; in fact, comics may become an effective means of education, as suggested by our preliminary study.

A deficiency in understanding health information may be, in large part, due to the inadequate way in which relevant information is conveyed (16–19). This inadequacy is particularly detrimental for those confronting communication barriers. Health communication and prevention has been challenged by the inequalities and disparities of those most in need of effective educational and preventive messages. Specifically, populations with reduced literacy, education, or language proficiency have reduced opportunities to benefit from traditional educational messages. This population is composed of minority groups, as well as young parents with low levels of education, literacy, and language proficiency. Despite the large amount of material developed to educate individuals confronting health disparities, the message is often not understood and learning is hindered or does not occur. Traditional approaches to solving this problem have failed due to inefficient delivery of the message (20).

This study has some limitations. Firstly, there was no control group. Additionally, a subset of parents could have been referring to the comic as they responded to the post-intervention questions when the telephone call was received, so they did not actually increase their knowledge during the post-intervention time period. Post-intervention responses were to be collected 4 weeks after the well-baby visit, but difficulty in contacting parents extended this period to 6 weeks for some parents. Moreover, parents could possibly recall the questions from the baseline test and find the answers from a different source, not necessarily from the educational comic. We attempted to overcome this potential limitation, however, by providing only 10 min to respond to the questions and by changing the question order in the post-intervention questionnaire to prevent the participants from remembering their responses. However, given that the correct responses were significantly different in the post-intervention test compared to baseline, it is possible that the variation in time to collect the responses did not have a negative influence on the results.

The possibilities of using this multimodal format as an educational tool for parents and children/adolescents should be further studied, and comics could be produced for each major milestone age group. Additionally, comics that address other important milestones in childhood/adolescent development (e.g., toilet training, puberty, starting school) and that discuss pathological conditions of childhood/adolescence (e.g., type 1 diabetes, inflammatory bowel disease, autism spectrum disorder) may merit further investigation.

The most interesting finding of this study is that caregivers took the comic, learned or reinforced their knowledge, and disseminated the information to others, allowing for a possible extended learning effect. The Bandura theory states that learning would be extremely laborious if people had to rely solely on the effects of their own actions to decide what to do in future scenarios. Fortunately, most human behavior is learned observationally through modeling. That is, a person may gather ideas and values by watching others in various situations and by learning from those experiences. This coded information then serves as a guide for action when similar situations are later encountered. The extraordinary capacity of symbolization provides humans with a powerful tool for comprehending their environment and creating and regulating environmental factors from virtually every aspect of their lives (21).

In relating the Bandura theory to our study, information was presented with a simple story and characters that allowed the observer to understand, relate to, and better retain the information presented. With this straightforward approach to learning, reinforced by concepts from the Bandura theory, it is possible that comics may become an alternative source for meaningful health education.

## Ethics statement

This Brief Research Report was eligible for exempt status.

## Author contributions

ML devised the project, the main conceptual ideas, and proof outline. ER-B verified the numerical results of the study. HG and BM proposed the presentation of article. ML, ER-B, HG, and BM wrote the manuscript.

### Conflict of interest statement

The authors declare that the research was conducted in the absence of any commercial or financial relationships that could be construed as a potential conflict of interest.
